# miR204 potentially promotes non-alcoholic fatty liver disease by inhibition of cpt1a in mouse hepatocytes

**DOI:** 10.1038/s42003-022-03945-1

**Published:** 2022-09-21

**Authors:** Seonhee Kim, Ikjun Lee, Shuyu Piao, Harsha Nagar, Su-jeong Choi, Young-Rae Kim, Kaikobad Irani, Byeong Hwa Jeon, Cuk-Seong Kim

**Affiliations:** 1 Department of Physiology & Medical Science, Chungnam National University College of Medicine, Daejeon, 35015 Republic of Korea; 2 Division of Cardiovascular Medicine, Department of Internal Medicine, University of Iowa Carver College of Medicine, Iowa City, IA 52242 USA

**Keywords:** Metabolic syndrome, Fat metabolism

## Abstract

Non-alcoholic fatty liver disease (NAFLD) is associated with hepatic metabolism dysfunction. However, the mechanistic role of miR204 in the development of NAFLD is unknown. We investigate the functional significance of miR204 in the evolution of NAFLD. IDH2 KO mice feed a normal diet (ND) or HFD increased body weight, epididymal fat-pad weight, lipid droplet in liver, blood parameter and inflammation compared to WT mice fed a ND or HFD. Moreover, the expression of miR204 is increased in mice with IDH2 deficiency. Increased miR204 by IDH2 deficiency regulates carnitine palmitoyltransferase 1a (cpt1a) synthesis, which inhibits fatty acid β-oxidation. Inhibition of miR204 prevents the disassembly of two fatty acid-related genes by activating CPT1a expression, which decreases lipid droplet in liver, inflammatory cytokines, epididymal fat pad weight, blood parameters. Increased miR204 by IDH2 deficiency promotes the pathogenesis of HFD-induced NAFLD by regulating hepatic fatty acid metabolism and inflammation.

## Abstract (web summary)

miR204 is found to inhibit cpt1a in mouse hepatocytes, which could play a role in promoting non-alcoholic fatty liver disease.

## Introduction

Metabolic disorders are rapidly increasing in incidence due to higher rates of obesity, diabetes, hyperlipidemia, and cardiovascular disease^1,2^. Non-alcoholic fatty liver disease (NAFLD) is a liver condition not caused by heavy alcohol consumption; its main characteristic is excess fat storage in liver cells. NAFLD is emerging as a major cause of chronic liver disease worldwide, accounting for 20% of cases globally^3^. Some patients with NAFLD develop non-alcoholic steatohepatitis (NASH), which is an aggressive form of fatty liver disease induced by liver inflammation that may progress to liver failure and cancer^4^.

Imbalance of energy homeostasis causes dysfunction of muscles, liver, and adipocytes, leading to metabolic disease. To remove excess energy, it is necessary to increase metabolic activity via fatty acid β-oxidation. Promoting the activation of lipid metabolism in mitochondria is essential^5,6^; mitochondrial dysfunction causes obesity-related disorders, but the underlying mechanisms are unclear^7,8^.

The mitochondrial protein, isocitrate dehydrogenase (IDH2) is essential for energy consumption, and converts the product of oxidative decarboxylation of isocitrate into α-ketoglutarate along with reduction of NADP^+^ to NADPH. IDH2 deficiency increases mitochondrial dysfunction and endothelial cell inflammation^9,10^. IDH2 deficiency induces the accumulation of citrate, which is transported from mitochondria to the cytosol^11^. We hypothesized that cytosol citrate could be converted into Acetyl-CoA, which is a precursor of fatty acids and cholesterol. IDH2 protects against the metabolic stress induced by a high-fat diet by limiting oxidative damage to the mitochondria in brown adipose tissue^12^. IDH2 knockout (KO) mice exhibit obesity resistance and insulin sensitivity^13^. However, there are no previous studies related to IDH2 deficiency and fat metabolism in the liver, where nutrients from the hepatic portal vein are synthesized into fat. Here, we examined the role of IDH2 deficiency in lipid metabolic disorder.

Carnitine palmitoyltransferase 1a (CPT1a) is a transporter of fatty acids for β-oxidation. This mitochondrial enzyme mediates the degradation of accumulated fatty acids for energy production in mitochondria. A decreased CPT1a level blocks fatty acid degeneration in mitochondria, resulting in intracellular accumulation of long-chain fatty acids. CPT1a overexpression protected against high-fat diet (HFD)-induced weight gain, insulin resistance, endoplasmic reticulum stress, inflammation, and liver damage^14^.

MicroRNAs (miRNAs) are small non-coding RNAs that regulate target gene expression and translation via binding to target mRNAs. miRNAs are expressed in various diseases, including metabolic disorders^15,16^. miR204 is abundant in damaged pancreatic β-cells^17^; however, whether it regulates metabolic disorders and obesity in vivo is unknown. miR204 regulates many functionally important β-cell genes. miR204 inhibition attenuates the HFD-triggered imbalance in glucose uptake^18,19^. We analyzed the hepatic miRNA profiles of IDH2 KO mice and wild-type (WT) mice. miR204 expression was more than threefold higher in IDH2 KO mice compared to WT mice.

In this study, we showed that miR204 was upregulated in steatotic hepatocytes and the livers of IDH2 KO mice as a NAFLD model. Elevation of miR204 in vitro and in vivo induced lipid accumulation and fatty acid synthesis in hepatocytes by destabilizing cpt1a mRNA to inhibit fatty acid β-oxidation. A miR204 inhibitor (miR204-I) significantly attenuated body weight gain, subcutaneous fat accumulation, and hepatic inflammation. Our findings reveal a novel post-transcriptional mechanism by which miR204 regulates CPT1a expression in the pathogenesis of NAFLD.

## Results

### IDH2 deficiency promotes HFD-induced NAFLD with obesity and inflammation in the liver

**Fig. 1:**
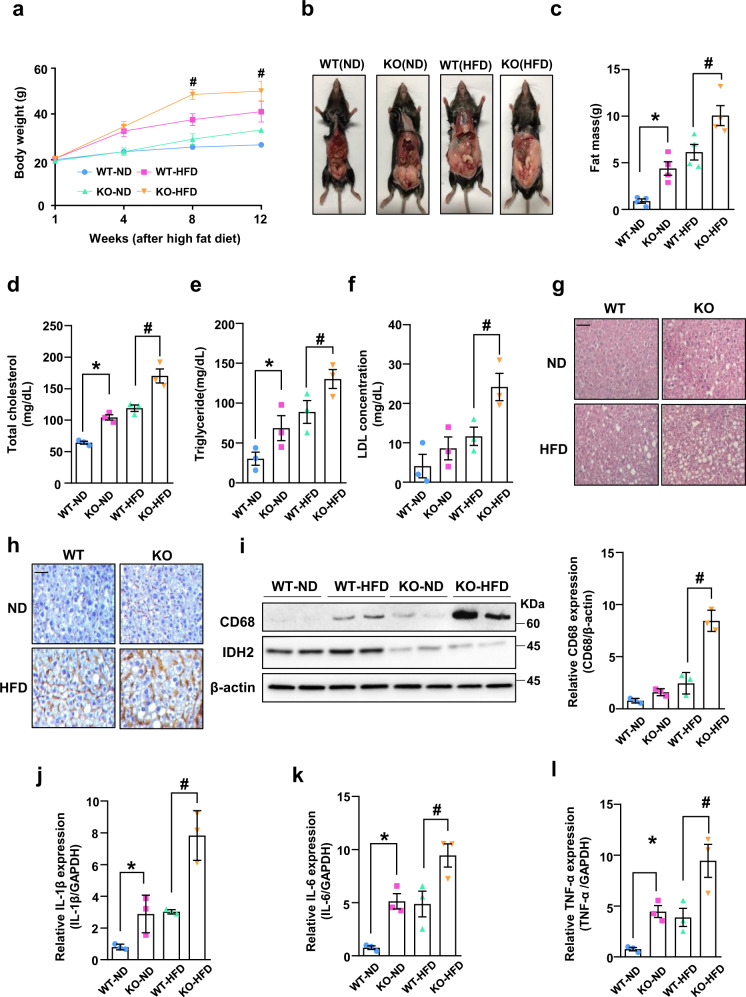
IDH2 deficiency increases fat mass and sensitivity to high-fat diet-induced obesity in NAFLD mice with inflammation of the liver. **a** Body weights of male mice fed a normal diet (ND, black-WT, green-KO) and high-fat diet (HFD, blue-WT, red-KO) for 1–12 weeks. **b** Photographs of WT and KO fed an ND or HFD. **c** Epididymal fat mass. **d**–**f** Blood parameters. Data are means ± SEM of three independent experiments. *p < 0.05 vs. WT-ND mice. #p < 0.05 *vs*. WT-HFD mice. **g** Representative H&E-stained high-magnification images of mouse livers. **h** Representative immunohistochemical images of CD68 in the liver of WT and KO mice fed an ND or HFD; scale bars, 50 μm. **i** Western blot analysis of CD68 in the liver tissues of two mice per group. **j**–**l** Expression of IL-1β (**j**), IL-6 (**k**), and TNF-α (**l**) by quantitative real-time PCR, normalized to GAPDH. Data are means ± SEM of three independent experiments (*n* = 3). *p < 0.05 vs. WT-ND mice. #*p* < 0.05 vs. WT-HFD mice.

To investigate the role of IDH2 in metabolic diseases characterized by tricarboxylic acid cycle (TCA) disorders, we compared the body weights of IDH2 KO and WT mice fed a normal diet (ND) or HFD. IDH2 KO mice fed an ND or HFD showed significantly increased body weight compared to WT mice (Fig. 1a, b). IDH2 KO mice fed an ND or HFD had epididymal fat pads about twice as large as those of WT mice (Fig. 1c). IDH2 KO mice showed significantly elevated serum levels of total cholesterol, triglycerides, and low-density lipoprotein (LDL) (Fig. 1d–f). Therefore, IDH2 KO mice fed an ND or HFD exhibited enhanced fat accumulation and cholesterol synthesis compared to WT mice.

Fatty acids absorbed in the small intestine are transported to the liver, where they are metabolized and used as an energy source, but excess fat intake induces metabolic disorders by causing hepatic fat accumulation. In addition, given the close relationships among obesity, abnormal lipid metabolism, and inflammation, we investigated the effect of IDH2 deficiency on lipid accumulation and inflammation in the liver. Histological analysis was performed to determine whether IDH2 deficiency induces metabolic disease. Hematoxylin-eosin (H&E) staining of liver sections showed greater hepatic lipid droplet accumulation in HFD-fed IDH2 KO mice compared to HFD-fed WT mice, and the same pattern was observed in ND-fed mice (Fig. 1g). Obesity contributes to activation and recruitment of inflammatory cells such as monocytes, neutrophils, and macrophages^20^. Chronic inflammation is responsible for the side effects of several metabolic diseases. Immunohistochemical staining for the macrophage marker CD68 was performed to assay macrophage infiltration in liver tissue. CD68 expression was significantly increased in HFD-fed IDH2 KO mice compared to HFD-fed WT mice (Fig. 1h). This finding was confirmed by western blotting (Fig. 1i). Under inflammatory conditions, macrophages secrete cytokines such as interleukin (IL)-1β, IL-6, and tumor necrosis factor (TNF)-α^21^, causing vascular permeability and recruitment of inflammatory cells. The cytokine levels were increased in IDH2 KO mice (Fig. 1j–l). Therefore, IDH2 deficiency induces fat accumulation and an inflammatory response in the liver.

### IDH2 deficiency upregulates fatty acid and cholesterol synthesis

**Fig. 2:**
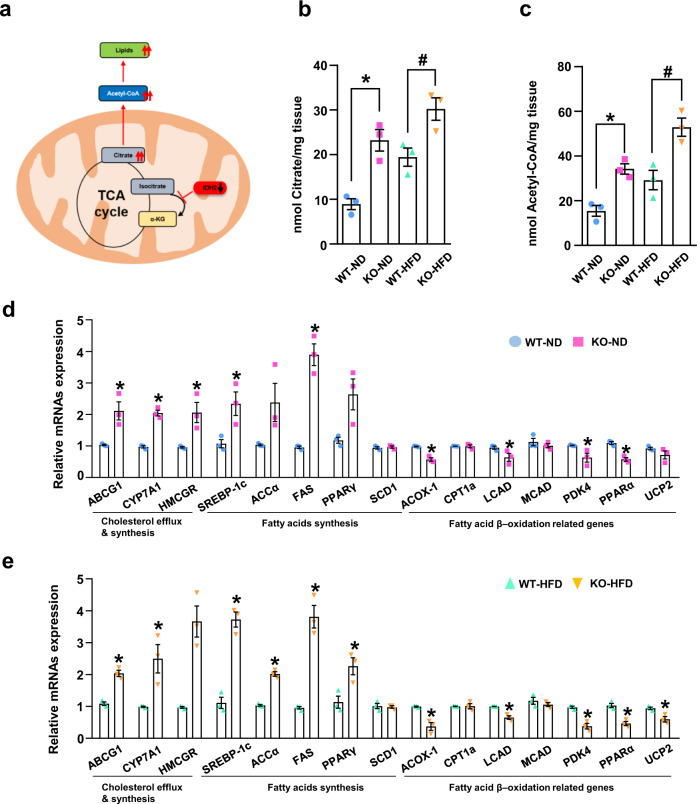
IDH2 deficiency upregulates fatty acid and cholesterol synthesis. **a** Mechanisms of fatty acid and cholesterol synthesis. **b**, **c** Citrate and acetyl-CoA levels in liver tissue determined using a colorimetric/fluorometric assay kit. **d**, **e** Expression of genes related to cholesterol efflux and synthesis, and fatty acid synthesis and β-oxidation, revealed by quantitative real-time PCR (normalized to GAPDH). Data are means ± SEM of three independent experiments (*n* = 3). *p < 0.05 vs. WT-ND mice. #*p* < 0.05 vs. WT-HFD mice.

IDH2 is a mitochondrial enzyme involved in the TCA cycle. In the absence of IDH2, citrate is not converted into α-KG in mitochondria. It was hypothesized that accumulated citrate exits the cytoplasm via channels and is converted into acetyl-CoA, thus promoting lipid synthesis (Fig. 2a). We evaluated the citrate and acetyl-CoA levels in liver tissue from IDH2 KO mice fed the ND or HFD (Fig. 2b, c). We also analyzed the mRNA levels of enzymes involved in lipid synthesis using acetyl-CoA as a precursor in IDH2 KO mice. The mRNA levels of ATP binding cassette subfamily G member 1 (ABCG1), cytochrome P450 family 7 subfamily A member 1 (CYP7A1), 3-hydroxy-3-methyl-glutaryl-coenzyme A reductase (HMCGR), and sterol regulatory element-binding protein 1c (SREBP-1c), which are involved in cholesterol synthesis, and acetyl-CoA carboxylase (ACCα), fatty acid synthase (FAS), and peroxisome proliferator-activated receptor γ (PPARγ), which are involved in fatty acid synthesis, were increased in IDH2 KO mice fed the ND or HFD. The mRNA levels of acyl-coenzyme A oxidase 1 (ACOX-1), long-chain acyl-CoA dehydrogenase (LCAD), peroxisome proliferator-activated receptor alpha (PPARα), pyruvate dehydrogenase kinase 4 (PDK4), and uncoupling protein 2 (UCP2), which are involved in fatty acid β-oxidation, were decreased in IDH2 mice compared to WT mice fed the ND or HFD (Fig. 2d, e). In summary, IDH2 KO results in accumulation of acetyl-CoA, which is converted into fatty acids and cholesterol, thereby suppressing fatty acid β-oxidation.

### IDH2 deficiency increases miR204 expression in the liver

**Fig. 3:**
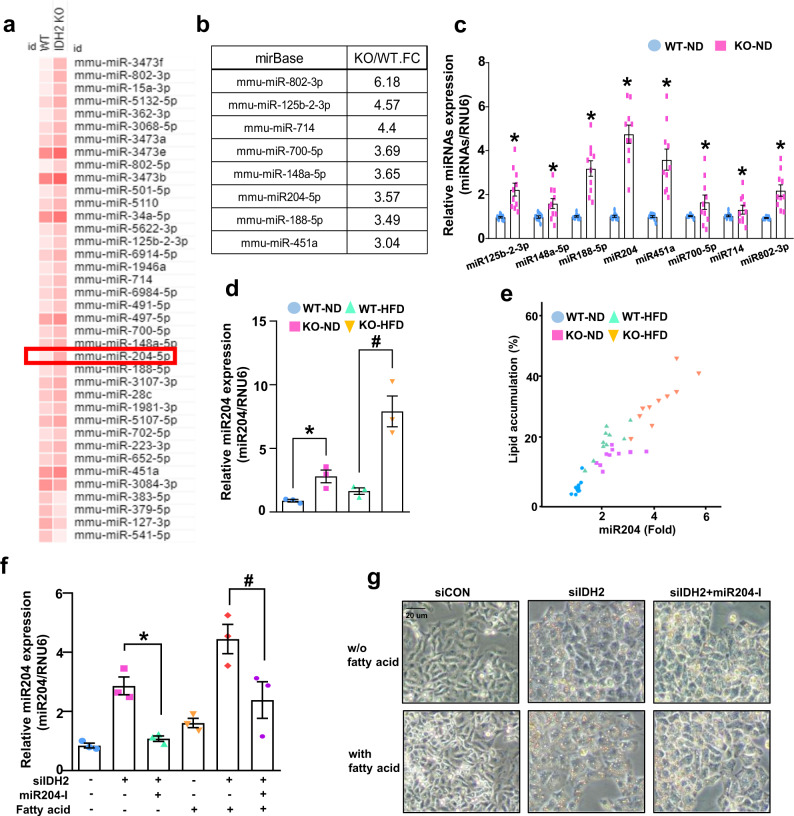
Correlation of lipid accumulation and miR204 expression in IDH2-deficient hepatocyte. **a** In total, 38 of 3163 miRNAs showed significant (at least threefold) up or downregulation. **b** Arrangement of fold values of eight miRNA array results reportedly linked to metabolic and cardiovascular diseases in IDH2 KO mice and WT mice liver. **c** Repeated by qPCR in the livers of WT and IDH2 KO mice from ten mice in each group **d** miR204 expression in mouse liver tissues revealed by qPCR. **e** Positive correlation of lipid accumulation and miR204 expression in liver. Pearson correlation coefficient (WT_ND: 0.51667, IDH2 KO_ND:0.28901096, WT_HFD:0.67427449, IDH2 KO_HFD:0.73240585). These cells were first transfected siCON, siIDH2 and miR204-I after 24 h, and then treated fatty acid 24 h. **f** miR204 expression in hepatocytes; RNU6 was used as the internal control. **g** Oil Red O staining of hepatocytes. Scale bars, 20 μm. Data are means ± SEM of ten independent experiments (*n* = 10). *p < 0.05 vs. WT-ND mice or control hepatocyte. #*p* < 0.05 vs. WT-HFD mice or siIDH2-transfected hepatocyte.

miRNAs are implicated in the development of NAFLD^22,23^. NAFLD is closely related to obesity, diabetes, and insulin resistance. Therefore, we investigated miRNA expression in HFD-induced obesity. The livers of WT and IDH2 KO mice were analyzed using the miRNA 4.0 array (Affymetrix, Santa Clara, CA, USA) by Macrogen (Seoul, South Korea). In total, 38 of 3,163 miRNAs were significantly (at least threefold) differentially regulated between the livers of WT and IDH2 KO mice (Fig. 3a). Among the 38 miRNAs, 8 miRNAs are reportedly associated with metabolic and cardiovascular diseases (miR-802-3p, miR125b-2-3p, miR714, miR700-5p, miR148a-5p, miR204-5p, miR-188-5p, and miR451a). We arranged the fold values of eight miRNAs in IDH2 KO mice and WT mice (Fig. 3b). To validate the sequencing data, the expression of these eight miRNAs was measured by quantitative PCR (qPCR) in the livers of WT and IDH2 KO mice from 10 mice in each group (Fig. 3c). We chose miR204 because of higher accuracy in these results, as compared to the sequencing data in Fig. 3a, b due to more number of mice used in the experiment. Generally, miRNAs regulate target gene expression by binding to the mRNA 3′UTR. The seed site is important for binding a miRNA to an mRNA. The seed site (or seed region) is a conserved heptametric sequence in mammals, which is typically situated 2–7 nucleotides from the 5′-end of the miRNA. miRNA significantly affects target gene mRNA expression in the 3′UTR. Although it is less effective than matching the 3′-UTR sequence, coding sequence (CDS) matching of miRNAs suppresses mRNA translation^24^. The top three miRNA-target genes (miR188-5p, miR204, and miR451a) were analyzed and matched to the CDS and 3′-UTR of genes with no mRNA alterations (stearoyl-CoA desaturase [SCD1], CPT1a, and medium-chain acyl-coenzyme [MCAD]) (Table 1). miR204 expression was increased in HFD-fed WT mice and IDH2 KO mice (Fig. 3d). Correlation of lipid accumulation and miR204 expression was detected through counting of droplet in H&E staining with miR204 qPCR results. As a result, we confirmed that there was a positive correlation using Pearson’s correlation coefficient between lipid accumulation and miR204 expression, and in the case of a high-fat diet, IDH2 deficiency further enhanced the correlation (Fig. 3e). We confirmed miR204 expression using miR204 inhibitor to confirm the association between miR204 and lipid synthesis also in vitro. An increase in miR204 was confirmed in fatty acid-treated hepatocytes (Fig. 3f), and as a result of controlling the expression of miR204 via a miR204 inhibitor, fat staining with oil red O decreased (Fig. 3g). Taken together, miR204 expression and lipid accumulation have a positive correlation, and that this relationship is further strengthened when IDH2 deficiency occurs.

**Table 1:**
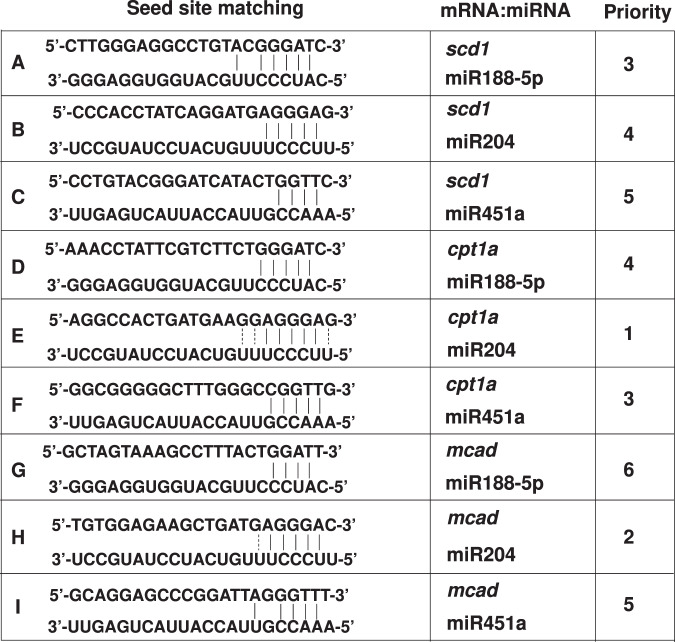
Sequence matching of miRNA-target mRNA interaction.

### miR204 inhibits the translation of CPT1a in vitro and in vivo

**Fig. 4:**
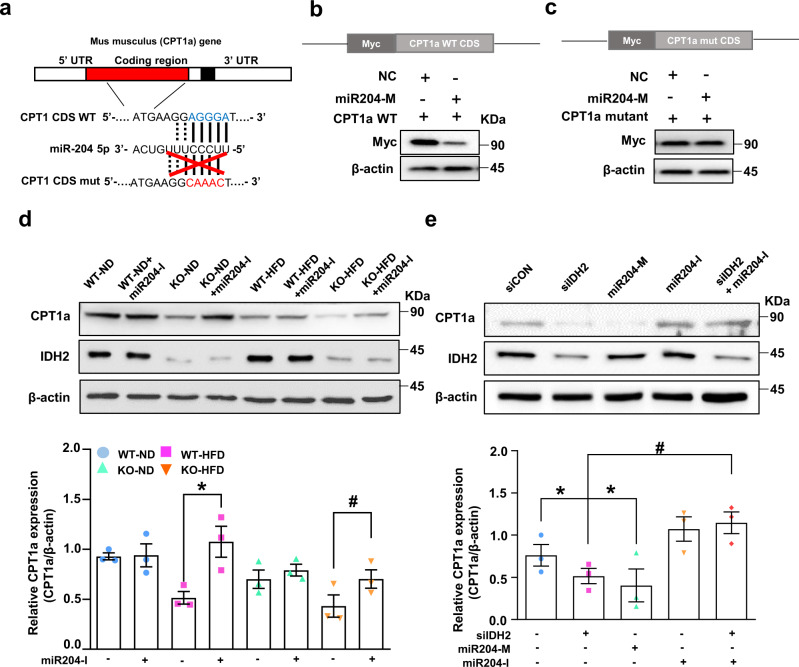
miR204 binds to the coding region of cpt1a mRNA and inhibits translation of CPT1a in mouse hepatocytes. **a** Expected binding site of cpt1a mRNA and miR204 from *Homo sapiens* and *Mus musculus*. **b** Overexpression of the miR204 duplex decreased the protein level of WT CPT1a. Construction of Myc-tagged WT CPT1a-coding region and western blots. **c** Overexpression of the miR204 duplex did not affect the protein level of mutant cpt1a. Construction of Myc-tagged and CPT1a mutated plasmid coding region and western blots. **d** Western blot analysis of CPT1a in ND- or HFD-fed WT and KO mice. **e** Western blot analysis of CPT1a in IDH2-deficient and miR204 inhibitor-treated mouse hepatocytes. β-actin was used as the internal control. Data are means ± SEM of three independent experiments (*n* = 3). $*p* < 0.05 vs. KO-ND mice. *p < 0.05 vs. siCON. #*p* < 0.05 vs. KO-HFD mice transfected with a miR204 mimic (204-M; overexpression).

To confirm direct targeting of CPT1a by miR204, we ectopically expressed the full-length CPT1a cDNA fused with a Myc-tag in hepatocytes. The CPT1a WT or mutated plasmid contained the predicted miR204 binding site (Fig. 4a). We used the cpt1a plasmid and a *Homo sapiens*-derived plasmid. The target sequence of miR204 was conserved and matched the seed site of the target mRNA of *Mus musculus*. CPT1a WT or the mutated plasmid was co-transfected with a miR204 mimic (204-M). Overexpression of miR204 inhibited Myc-CPT1a expression in the CPT1a WT plasmid only, but this effect was not seen with mutant plasmid transfection (Fig. 4b, c). A miR204 inhibitor rescued CPT1a expression, which was reduced by IDH2 deletion in ND- or HFD-fed mouse liver tissues and hepatocytes (Fig. 4d, e). Therefore, the increased miR204 expression caused by IDH2 deficiency regulates CPT1a protein synthesis.

### Inhibition of miR204 ameliorates fatty acid β-oxidation in the hepatocytes

**Fig. 5:**
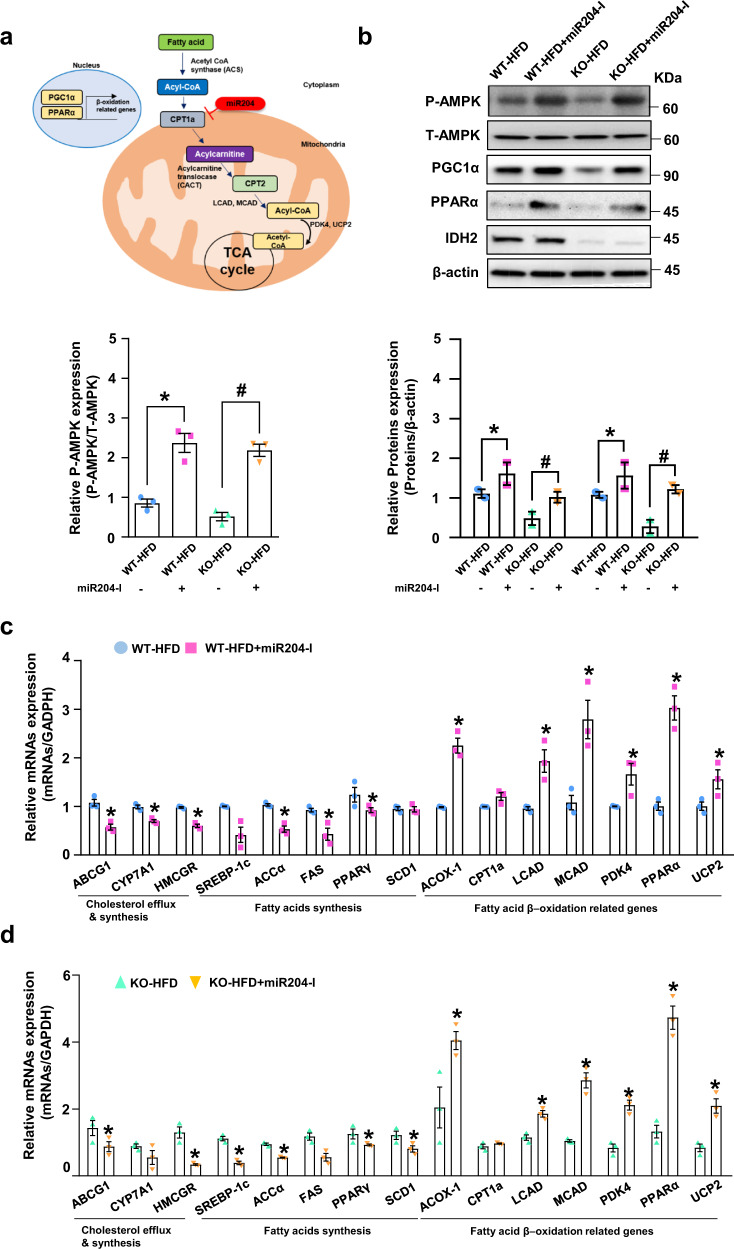
Inhibition of miR204 ameliorates fatty acid β-oxidation in liver. **a** Degradation of fatty acids. **b** Western blot analysis of the phosphorylation of AMPK, PGC1α, and PPARα in the liver of IDH2 KO and WT mice fed an HFD, and treated or not with an miR204 inhibitor. β-actin was used as the internal control. **c**, **d** Expression of genes related to cholesterol efflux and synthesis, fatty acid synthesis, and fatty acid β-oxidation in the liver of IDH2 KO and WT mice fed an HFD, and treated or not with a miR204 inhibitor, as determined by quantitative real-time PCR (normalized to GAPDH). Data are means ± SEM of three independent experiments (*n* = 3). *p < 0.05 vs. WT-HFD mice. #p < 0.05 vs. KO-HFD mice.

Fatty acid β-oxidation is the process by which fatty acids are broken down to generate energy. The increased miR204 expression caused by IDH2 downregulation inhibits CPT1a, a key regulator of fatty acid β-oxidation in mitochondria (Fig. 5a). The expression levels of the upstream markers of fatty acid β-oxidation—AMP-activated protein kinase (AMPK) phosphorylation, peroxisome proliferator-activated receptor gamma coactivator-1 alpha (PGC1α), and PPARα—were restored in miR204-I-injected WT and IDH2 KO mice (Fig. 5b). The mRNA levels of enzymes involved in fatty acid β-oxidation were confirmed in IDH2 KO mice and WT mice injected with miR204-I (Fig. 5c, d). CPT1a restoration rescued the levels of downstream molecules related to fatty acid β-oxidation, such as LCAD, MCAD, and UCP2. In vitro, we measured the fatty acid oxidation (FAO) activity by ^18^C-labeled unsaturated fatty acid oleate as the substrates with CPT1a inhibitor, Etomoxir (ETX) and the FAO activator FCCP as negative and positive controls, respectively. The FAO was decreased by IDH2 deficiency, however, inhibition of miR204 rescues FAO activity in IDH2-deficient hepatocytes (Supplementary Fig. 2). Therefore, miR204 blocks the degradation of fatty acids, leading to the accumulation of fat and cholesterol in the hepatocytes.

### miR204 inhibition attenuates IDH2 deletion-induced NAFLD

**Fig. 6:**
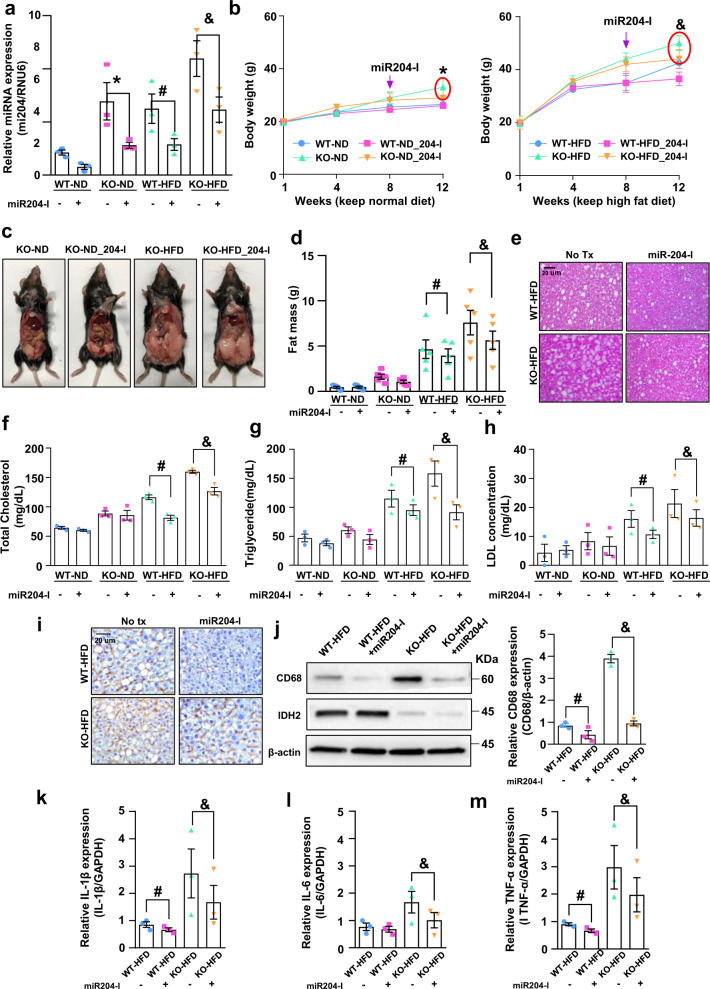
miR204 inhibition attenuates IDH2 deletion-induced NAFLD. **a** miR204 expression in liver tissues; RNU6 was used as the internal control. **b** Body weight of male mice injected with miR204-I and fed the ND (black-WT, blue-WT + 204-I, green-KO, red-KO + 204-I) or HFD (black-WT, blue-WT + 204-I, green-KO, red-KO + 204-I) at 1–12 weeks. **c** Photographs of KO mice treated or not with an miR204-I and fed an ND or HFD. **d** Epididymal fat mass. **e** Representative H&E-stained high-magnification images of mouse livers; scale bars, 50 μm. **f**–**h** Blood parameters. **i** Representative immunohistochemical images of CD68 in liver tissues of WT and KO-HFD-fed mice treated or not with miR204-I. Scale bars, 50 μm. **j** Western blot analysis of CD68 in liver tissues of two mice per group. **k**–**m** Expression of IL-1β (**k**), IL-6 (**l**), and TNF-α (**m**) by quantitative real-time PCR, normalized to GAPDH. Data are means ± SEM of three independent experiments (*n* = 3). *p < 0.05 vs. KO-ND mice. #p < 0.05 vs. WT-HFD mice. &*p* < 0.05 vs. KO-HFD mice.

To assess the role of miR204 in the development of fatty liver and obesity in IDH2 KO mice, we compared subcutaneous fat accumulation and blood parameters between WT and KO mice fed an ND or HFD for 12 weeks. miR204 inhibitors were injected subcutaneously (s.c.) using an osmotic pump for 4 weeks after feeding with the HFD for 8 weeks. Inhibition of miR204 significantly decreased the hepatic miR204 level (Fig. 6a). Inhibition of miR204 suppressed the increase in body weight of KO mice fed an ND or HFD (Fig. 6b, c). Inhibition of miR204 reduced the epididymal fat-pad weight in WT and KO mice fed an ND or HFD (Fig. 6d). Also, miR204 inhibition significantly rescued excessive lipid accumulation, as determined by H&E staining (Fig. 6e) and analysis of the serum levels of total cholesterol, triglyceride and LDL in WT and KO mice fed an HFD (Fig. 6f–h). Therefore, miR204 inhibition activates β-oxidation of fatty acid by restoring CPT1a expression in the liver.

The CD68 immunohistochemical signal intensity was decreased by miR204-I (Fig. 6i). Western blotting also showed that inhibition of miR204 rescued macrophage infiltration in the liver (Fig. 6j). Additionally, miR204-I-treated KO-HFD mice exhibited lower IL-1β, IL-6, and TNF-α mRNA levels than KO-HFD mice (Fig. 6k–m). These findings suggest that miR204 inhibition attenuates inflammation by inhibiting the synthesis of pro-inflammatory cytokines in KO mice.

## Discussion

The prevalence of NAFLD has increased significantly in most developed countries over the past few decades. NAFLD involves low-level inflammation in insulin-targeted tissues such as muscle, fat, and liver^25^. Chronic inflammation increases the levels of chemokines and cytokines, inducing systemic embolic disorder in the liver^26^. Mitochondrial dysfunction promoted the development of metabolic diseases by modulating the level of active oxygen, the reducing power of oxygen radicals, and cellular energy production^12^. Maintaining mitochondrial homeostasis is a prerequisite for preventing metabolic diseases.

Deficiency of the mitochondrial protein IDH2 induces mitochondrial dysfunction, inflammation, and endothelium-dependent vasomotor function in endothelial cells^9,10^. In this study, IDH2 KO mice fed an ND or HFD exhibited severe fat accumulation, and elevated blood cholesterol and triglyceride levels, compared to WT mice (Fig. 1). Notably, IDH2 KO mice fed an HFD exhibited significantly increased levels of pro-inflammatory cytokines, and the expression of fatty acid and cholesterol efflux related genes (both of which regulate inflammation and lipid metabolism), compared to WT mice fed an HFD (Figs. 1 and 2).

IDH2 deletion increased obesity resistance and metabolic stress through brown adipose tissue whitening^12,13^. Therefore, we used IDH2 KO mice for NAFLD studies, assuming that IDH2 KO mice with HFD more accelerates the phenomenon of fat accumulation than WT mice with HFD. In addition, we investigated the direct mechanism of fat accumulation in the liver. IDH2 deficiency increased hepatic miR204 expression (Fig. 3). miR204 regulates cardiovascular diseases by modulating SIRT1 expression^27,28^, suppresses tumor-cell proliferation^29^ and regulates pancreatic β-cell proliferation^30,31^. We hypothesized that the increased miR204 expression caused by IDH2 downregulation regulates genes related to lipid metabolism. miRNAs inhibit gene expression by direct post-transcriptional gene silencing in the cytosol, achieved by binding to the 3′-UTR of the mRNA^32^ without altering target gene expression. However, miRNAs also inhibit target gene translation by directly binding the CDS^24^. The mRNA levels of MCAD, SCD1 and CPT1a were similar between ND and HFD IDH2 KO mice. The CDS of CPT1a was prioritized over other candidates to match the seed site of miR204 (Fig. 4). Also, there was no change in Myc expression in CPT1a mutant plasmid-transfected cells, indicating that miR204 inhibits CPT1a protein synthesis by matching the target sequence in the CDS of CPT1a (Fig. 4). A more important finding in this study was to clarify the correlation between liver fat accumulation due to IDH2 deficiency and miR204 in a high-fat diet (Pearson correlation coefficient) (Fig. 3d and Supplementary Fig. 1).

AMPK plays a central role in the regulation of energy homeostasis in the body, as well as fat metabolism^33^. Abnormalities in these energy sensors are related to the development of cardiovascular diseases, metabolic disorders, and cancer. Upon activation, AMPK suppresses the synthesis of fatty acids and cholesterol, and promotes the oxidation of fatty acids. HFD-fed IDH2 KO mice showed suppressed AMPK activation (phosphorylation) compared to HFD-fed WT mice (Fig. 5b). An important regulatory mechanism of AMPK is the inhibition via phosphorylation of acetyl-CoA carboxylase (ACC)^34,35^. ACC regulates lipid metabolism in multiple tissues, such as the liver and muscle, and ACC transforms acetyl-CoA into malonyl-CoA. Malonyl-CoA is present in the outer membrane of mitochondria and inhibits CPT1a. CPT1a plays an important role in β-oxidation in mitochondria. As expected, inhibition of miR204 attenuated fat accumulation and inflammation in this study, as indicated by the blood levels of several lipid markers and body and fat-pad weights in WT and KO mice (Fig. 6).

Our data demonstrate that the increased miR204 level caused by IDH2 deficiency modulates lipid metabolism by inhibiting CPT1a expression. Whitening of adipose tissue and mitochondrial dysfunction of adipocytes due to IDH2 deficiency are well known^12^. However, the effect of IDH2 deficiency on hepatocytes has not yet been reported. IDH2 deficiency causes citrate accumulation, which could be converted to Acetyl-CoA, and it can be a precursor to fatty acid synthesis in hepatocytes (Fig. 2b, c). In addition, there is no change in miR204 in WT-HFD mice compared to WT-ND (Fig. 3d). However, miR204 inhibition affects amelioration of NAFLD in WT-HFD (Figs. 4–6). As the deficiency of IDH2 and the effect of miR204 inhibitor affected not only hepatocytes but also cells of some organs (e.g., pancreatic beta cells, brown fat). We plan to enhance the originality of the disease mechanism through hepatocyte-specific IDH2 & miR204 double-deficient mice and study the association of miR204 / IDH2 in samples obtained from patients with non-alcoholic fatty liver disease. Despite the ever-increasing burden of NAFLD, it remains an unmet medical need with no approved pharmacologic treatment. Hence, strategies to regulate miR204 may have therapeutic potential for NAFLD and other liver diseases linked to mitochondrial dysfunction.

## Methods

### Cell culture and transfection

Mouse hepatocytes were purchased from the American Type Tissue Collection (Cat. CRL-2390; ATCC, Manassas, VA, USA) and cultured in F-12K medium (ATCC; Cat. #30-2004) containing 10% fetal bovine serum (Cat. #F-0500-A) (Atlas Biologicals, Fort Collins, CO, USA) and 1% antibiotic-antimycotic (Cat. #15240096; Gibco, Thermo Fisher Scientific, Inc., Waltham, MA, USA) at 37 °C in 5% CO_2_ according to the manufacturer’s instructions. Hepatocytes were transfected with an siRNA targeting IDH2 (mouse siRNA sequence: sense, 5′-GCGACCAGUACAAGGCCACAGAUUU-3′; antisense, 5′-AAAUCUGUGGCCUUGUACUGGUCGC-3′) and a negative control siRNA using Lipofectamine 2000 (Invitrogen, Carlsbad, CA, USA) according to the manufacturer’s instructions. For miR204 regulation, hepatocytes were transfected with miR204-I (5′-AGG ATG ACA AAG GGA-3′), a miR204 mimic sequence, or a scrambled nucleotide (5′-ACG TCT ATA CGC CCA-3′) using Lipofectamine 2000 (Invitrogen) according to the manufacturer’s instructions. For gene overexpression, hepatocytes were transfected with Myc-cpt1a (Cat. #100146; Addgene, Cambridge, MA, USA) full-length plasmid using Lipofectamine 2000 (Invitrogen) according to the manufacturer’s recommendations. Transfected cells were incubated for 2 days at 37 °C in a 5% CO_2_ incubator.

### Mouse studies

Experiments involving mice were approved by and followed the guidelines of the Institutional Animal Care and Use Committee at Chungnam National University Hospital (CNUH-019-A0016). The animals used were IDH2^−/−^ germ-line KO mice, and their congenic background strain (C57BL/6J) served as the WT (IDH2^+/+^) control. Mice were maintained in a controlled environment (ambient temperature, 22–24 °C; humidity, 50–60%; 12 h light/dark cycle). IDH2 KO mice were a gift from Dr. Jeen-Woo Park (School of Life Sciences and Biotechnology, College of Natural Science, Kyungpook National University, South Korea). An HFD (60% of total calories come from fat, Teklad Custom Diet (Cat. TD.06414)-full composition: [Casein 265.0 g/kg, l-Cystine 4 g/kg, Maltodextrin 160 g/kg, Sucrose 90 g/kg, Lard 310 g/kg, Soybean Oil 30 g/kg, Cellulose 65.5 g/kg, Mineral Mix (AIN-93G-MX, 94046) 48 g/kg, Calcium Phosphate, dibasic 3.4 g/kg, Vitamin Mix (AIN-93-VX, 94047) 21.0 g/kg, Choline Bitartrate 3.0 g/kg]) was fed to WT male mice and IDH2 KO male mice (8 weeks old) for 12 weeks (Doo Yeol Biotech, Seoul, South Korea).

### Immunoblotting

Cultured hepatocytes and liver tissues were harvested and lysed in 100 µl of RIPA buffer (1% NP-40, 0.5% Na-deoxycholate, 0.1% SDS, 0.15 M NaCl, 0.05 M Tris-HCl; pH 8), containing 1× Halt protease inhibitor cocktail (Thermo Fisher Scientific) for 30 min on ice. Liver tissue samples were then homogenized for 3 min in RIPA buffer on ice. After clearing by centrifugation at 13,000 rpm for 10 min, the protein concentration was measured using a bicinchoninic acid (BCA) protein assay kit (Thermo Fisher Scientific). Equal amounts of protein per well were resolved by 10–12% sodium dodecyl sulfate-polyacrylamide gel electrophoresis (SDS-PAGE) and transferred onto a nitrocellulose membrane. The membrane was washed with Tris-buffered saline (10 mM Tris, 150 mM NaCl) containing 0.1% Tween 20 (TBST) and blocked in TBST containing 5% bovine serum albumin Fraction V (Roche, Basel, Switzerland). Membranes were then incubated with the following antibodies: anti-IDH2 (ab129180), anti-CD68 (ab125212), anti-CPT1a (ab83862), anti-PPAR alpha (ab24509) (Abcam, Cambridge, UK); anti-PGC1α (sc-13067) (Santa Cruz Biotechnology, Santa Cruz, CA, USA); anti-phospho-AMPK (2531S), anti-AMPK (2532S) (Cell Signaling Technology, Danvers, MA, USA); and anti-β-actin (A-5441) (Sigma-Aldrich, St. Louis, MO, USA). Immunoblotting of 30 μg of whole-cell lysate or tissue homogenate was performed using appropriate primary and horseradish peroxidase (HRP)-conjugated secondary antibodies, and the chemiluminescent signal was developed using Super Signal West Pico or Femto Substrate (Pierce Biotechnology, Rockford, IL, USA). Values were normalized to β-actin as a loading control.

### Real-time qPCR

Total RNA was isolated using TRIzol Reagent (Invitrogen) based on the acid guanidinium thiocyanate–phenol–chloroform method. The total RNA concentration was determined using a SmartSpec 3000 spectrophotometer (Bio-Rad, Hercules, CA, USA). Complementary DNA was prepared from total RNA using the Maxime RT Premix kit (iNtRON Biotechnology, Seongnam, Korea)). Quantitative real-time PCR was performed using the Prism7000 Sequence Detection System (Applied Biosystems, Foster City, CA, USA) with the Super Script III Platinum SYBR GreenOne-Step qRT-PCR Kit (Invitrogen). The primers used for mouse ABCG1 were sense 5′-GAT TGG CTT CAG GAT GTC CAT GTT GGA A-3′ and antisense 5′-GTA TTT TTG CAA GGC TAC CAG TTA CAT TTG ACA A-3′; those for mouse CYP7A1 were sense 5′-CTG TGT TCA CTT TCT GAA GCC ATG-3′ and antisense 5′-CCC AGG CAT TGC TCT TTG AT-3′; those for mouse HMCGR and mouse SREBP-1c were sense 5′-AGG GGT AGG GCC AAC GGC CT-3′ and antisense 5′- CAA GCT GCC TGG GGA GCT GGT A-3′; those for mouse ACCa were sense 5′ -GGC CAG TGC TAT GCT GAG AT-3′ and antisense 5′ AGG GTC AAG TGC TGC TCC A-3′; those for mouse FAS were sense 5′-CTG CGG AAA CTT CAG GAA ATG-3′ and antisense 5′-GGT TCG GAA TGC TAT CCA GG-3′; those for mouse PPARγ were sense 5′-CAA GAC TAC CCT TTA CTG AA-3′ and antisense 5′-CTA CTT TGA TCG CAC TTT GGT-3′; those for mouse SCD1 were sense 5′-CTT CTT GCG ATA CAC TCT GG-3′ and antisense 5′-TGA ATG TTC TTG TCG TAG GG-3′; those for mouse ACOX-1 were sense 5′-GAA TTT GGC ATC GCA GAC CC-3′ and antisense 5′-GAT CTC CAG ATT CCA GGC CG-3′; those for mouse CPT1a were sense 5′-TCT TGC AGT CGA CTC ACC TT-3′ and antisense 5′-TCC ACA GGA CAC ATA GTC AGG-3′; those for mouse LCAD were sense 5′-AAG GAT TTA TTA AGG GCA AGA AGC-3′ and antisense 5′-GGA AGC GGA GGC GGA GTC-3′; those for mouse MCAD were sense 5′-GAG CCT GGG AAC TCG GCT TGA-3′ and antisense 5′-GCC AAG GCC ACC GCA ACT TT-3′; those for mouse PDK4 were sense 5′-TGT GAT GTG GTA GCA GTA GTC-3′ and antisense 5′-GTG GTG AAG GTG TGA AGG A-3′; those for mouse PPARα were sense 5′-CAA GGC CTC AGG GTA CCA CT-3′ and antisense 5-‘TTG CAG CTC CGA TCA CAC TT-3′; those for mouse UCP2 were sense 5′-GTT CCT CTG TCT CGT CTT GC-3′ and antisense 5′-GGC CTT GAA ACC AAC CA-3′; and those for mouse GAPDH were sense 5′-GGT GAA GGT CGG TGT GAA CG-3′ and antisense 5′-CCC GTA GGG CGA TTA CAG TC-3′ (internal control). Dissociation curves were generated to check for primer dimers. Fold-changes in gene expression were calculated by the 2^–ΔΔCt^ method.

### miRNA isolation and qPCR

Total RNA was isolated using TRIzol Reagent (Invitrogen) based on the acid guanidinium thiocyanate–phenol–chloroform method. The total RNA concentration was determined using a SmartSpec 3000 spectrophotometer (Bio-Rad). Complementary DNA was prepared from total RNA using the miScript II RT Kit (Cat. #218161; Qiagen, Hilden, Germany). Quantitative real-time PCR was performed using the Prism7000 Sequence Detection System (Applied Biosystems) with the miScript SYBR Green PCR Kit (Cat. #218073; Qiagen). The primer used for mmu-miR204 was sense 5′-CGC TTC CCT TTG TCA TCC TA-3′; mmu-miR125-2-3p was sense, 5′-GTCAGGCTCTTGGGACAAAA-3′; mmu-miR148a-5p was sense, 5′-GTTCTGAGACACTCCGACTAAAA-3′; mmu-miR188-5p was sense, 5′-CATCCCTTGCATGGTGGAG-3′; mmu-miR451a was sense, 5′-CGAAAACCGTTACCATTACTGA-3′; mmu-miR700-5p was sense, 5′-GCTTACAATCTAGCTGGGAAAAA-3′; mmu-miR714 was sense, 5′-CCCAGGGAGAGACGTAAGAA-3′; mmu-miR802-3p was sense, 5′-CGCAGTAACAAAGATTCATCCTTGTAAAA-3′ and the antisense primer was a universal primer included in the SYBR Green PCR kit. The primer used for RNU6 was sense 5′-GCA AAT TCG TGA AGC GTT CC-3′ and the antisense primer was a universal primer included in the SYBR Green PCR Kit (internal control). Dissociation curves were generated to check for primer dimers. Fold-changes in gene expression were calculated by the 2^−ΔΔCt^ method.

### Histological analysis

After washing with phosphate-buffered saline, tumor tissues were fixed in 4% (w/v) paraformaldehyde and embedded in paraffin. Paraffin sections were deparaffinized and rehydrated according to standard protocols and stained with H&E. For immunohistochemistry, tumor tissues were stained with an anti-CD68 primary antibody (diluted 1:100; Cat. #ab125212; Abcam) overnight at 4 °C. Next, an HRP-conjugated anti-rabbit IgG was added for 60 min at room temperature. Color was developed by incubation for 5 s with 3,3′-diaminobenzidine (DAB). Sections were counterstained with hematoxylin and examined under a microscope (Motic, Richmond, BC, Canada) at 100× magnification.

### In vivo inhibition of miR204

An inhibitor of miR204 (5′-AGG ATG ACA AAG GGA-3′; miR204-I) (Bioneer, Daejeon, South Korea) was systemically infused into mice for 4 weeks (0.5 mg/kg/day) using an ALZET Osmotic Pump (Cat. 1004) implanted subcutaneously.

### Citrate assay

Livers were extracted and homogenized in citrate assay buffer. The citrate concentration was determined using a Citrate Colorimetric/Fluorometric Assay Kit (Cat. #K655-100; Biovision, Milpitas, CA, USA) according to the manufacturer’s instructions.

### Acetyl-CoA assay

Livers were extracted and homogenized in acetyl-CoA assay buffer. The acetyl-CoA concentration was determined using a PicoProbe™ Acetyl-CoA Colorimetric/Fluorometric Assay Kit (Cat. #K317; Biovision) according to the manufacturer’s instructions.

### Blood analysis

Blood parameters (glucose, cholesterol, triglycerides, high-density lipoprotein, and LDL) were analyzed using a Samsung LABGEO^PT^ Lipid Test 5 Kit with a Samsung LABGEO PT10 Analyzer according to the manufacturer’s instructions.

### Fatty acid in vitro treatment and Oil Red O staining

After 24 h transfection, these cells treated fatty acid (1 mM: Oleic acid 0.66 mM and Palmitic acid 0.33 mM) with cell growth media and then incubation for 24 h. Cells were then washed three times with ice PBS and fixed in 4% Paraformaldehyde 30 min at room temperature. After fixation, cells were washed three times with PBS and stained Oil red O solution (working solution, 0.5 g Oil Red O powder dissolved in 60% isopropanol) for 30 min at room temperature. Cells were washed again with PBS to remove unbound staining. To quantify Oil Red O content levels, dimethyl sulfoxide was added to each sample; after shaking at room temperature for 5 min and then were analyzed using a microscope.

### Fatty acid oxidation (FAO) assay

FAO assay was following the manufacturer’s instruction of Fatty Oxidation Assay Kit (Cat. ab217602, Abcam). Hepatocyte 1 × 10^4^ cells were seeded per well in 96 well plates and growing overnight. The cells transfected with siCON, siIDH2 and siIDH2 + miR204 inhibitor three wells per condition. The wells without cells were used as signal control. After 24 h, the cells rinsed twice with 100 μl pre-warmed FA-Free media followed by adding 90 μl pre-warmed FA measurement Medium. A total of 85 μl of FA-Free measurement medium was added to the wells, and 5 μl of BSA control were included as the FA-Free control. All wells except the signal control had 10 μl extracellular oxygen consumption reagent added from Extracellular Oxygen Consumption Assay Kit (Cat. Ab197243, Abcam). FCCP (0.625 μM, FAO activator) and Etomoxir (40 μM, CPT1a inhibitor, FAO inhibitor) added were included as signal control. And then the cells were sealed with 100 μl pre-warmed mineral oil, and the FAO signal were collected at 5 min intervals for 90 min at Ex/Em = 380/650 nm. The proteins were normalized by protein concentration using the BCA assay.

### Statistical analysis

Statistical analysis was performed using Prism 8 software (GraphPad Software Inc., La Jolla, CA, USA). Data are means ± standard error of the mean (SEM). Differences between two groups were evaluated using t-tests. For multiple comparisons, one-way analysis of variance (ANOVA) was performed followed by a Tukey’s multiple comparison test. *p*-values < 0.05 were considered indicative of statistical significance. Data are representative of at least three independent experiments.

### Reporting summary

Further information on research design is available in the Nature Research Reporting Summary linked to this article.

## Supplementary information

Supplementary Information

Reporting Summary

## Data Availability

The datasets supporting the conclusions of this article are available from the corresponding author upon reasonable request.Original data related to this work: Kim, Seonhee; Kim, Cuk-Seong (2022), Western blot quantification, Dryad, Dataset, 10.5061/dryad.crjdfn36h. Unprocessed data Sharing Link: https://datadryad.org/stash/share/ClKxEuHhpvKsX722hq4doBx7qNqneRCiX4ZhD5y4J70. Uncropped and unedited blot/gel images are listed in Supplementary Fig. 3.
