# Supplementary material for: miR204 potentially promotes non-alcoholic fatty liver disease by inhibition of cpt1a in mouse hepatocytes
Source: Commun Biol. 2022 Sep 21;5:1002. doi: 10.1038/s42003-022-03945-1 (PMC9492679; doi:10.1038/s42003-022-03945-1)
Supplement: Supplementary file 1 — Supplementary Information [file 42003_2022_3945_MOESM1_ESM.pdf]

Supplementary Fig. 1 Correlation of lipid accumulation and miR204 expression in IDH2 deleted hepatocytes with 1 mM fatty acid (Oleic acid 0.66 mM : palmitic acid 0.33 mM).

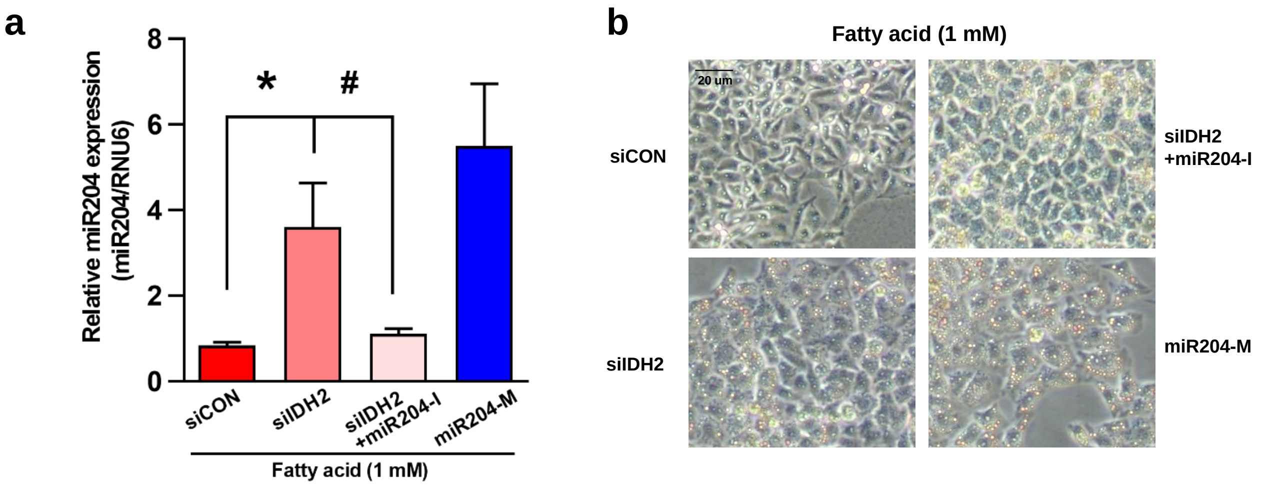

These cells were first transfected siCON, siIDH2, miR204-I and miR204-M after 24 h, and then treated fatty acid 24 h. **a** miR204 expression in hepatocytes; RNU6 was used as the internal control. **b** Oil Red O staining of hepatocytes. Scale bars, 20 μm. Data are means ± SEM of three independent experiments. *p < 0.05 *vs.* control hepatocyte. #p < 0.05 *vs.* siIDH2 transfected hepatocyte.

Supplementary Fig. 2 Inhibition of miR204 rescues Fatty Acid Oxidation (FAO) in IDH2 deficient hepatocytes

a

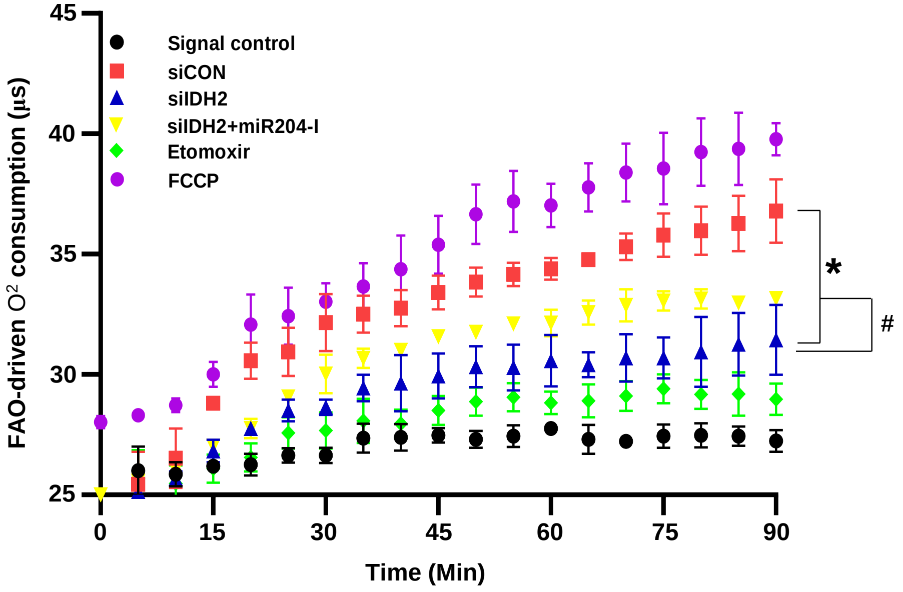

These cells were first transfected siCON, siIDH2 and siIDH2+miR204 after 24 h, and then measured FAO. **a** FAO activity in hepatocytes; FCCP and Etomoxir were used as the positive control and Negative control in FAO activity, respectively. Data are means ± SEM of three independent experiments. *p < 0.05 *vs*. siCON transfected hepatocyte. #p < 0.05 *vs*. siIDH2 transfected hepatocyte.

Supplementary Fig. 3 Uncropped and unedited blot/gel images

Fig.1I

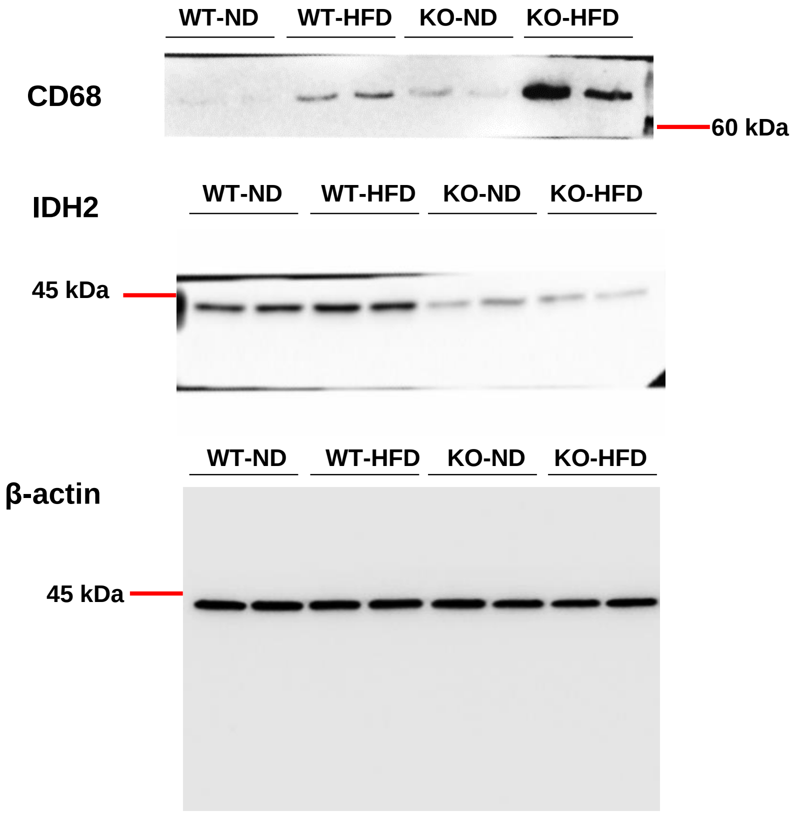

Fig.4D

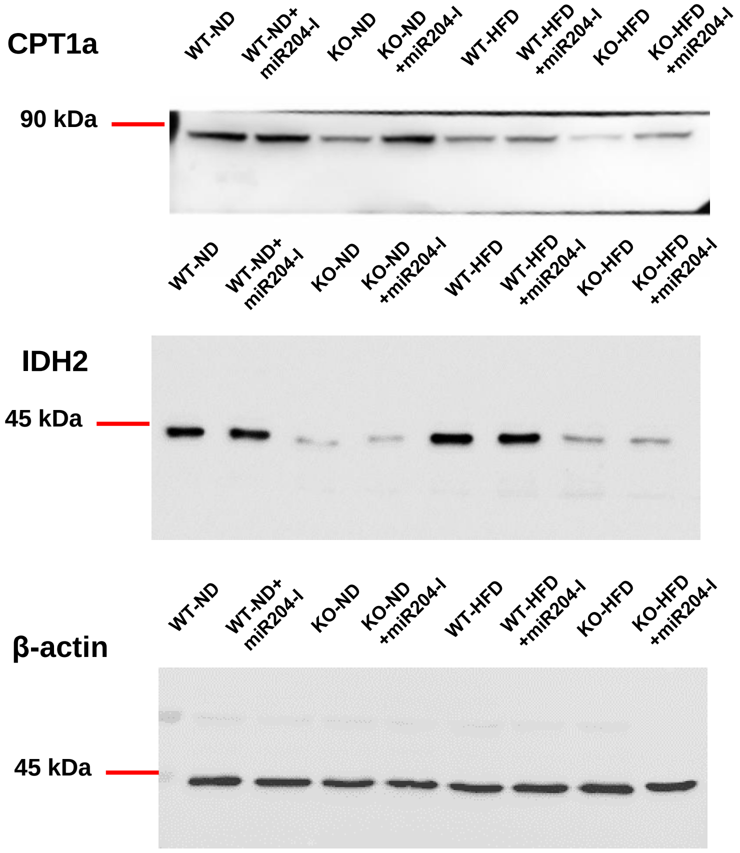

Fig.4E

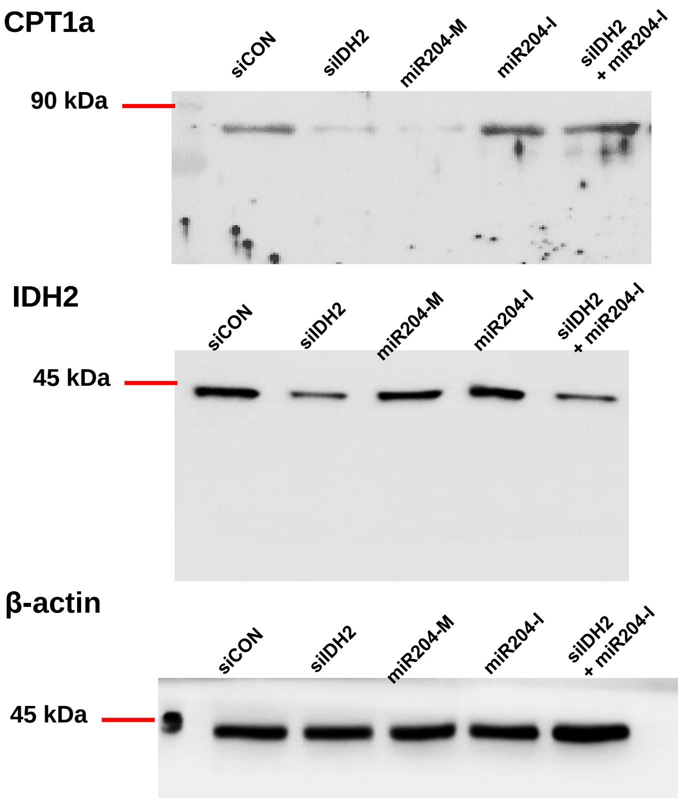
CPT1a
siCON
siIDH2
miR204-M
miR204-I
siIDH2
+ miR204-I
90 kDa
IDH2
siCON
siIDH2
miR204-M
miR204-I
siIDH2
+ miR204-I
45 kDa
β-actin
siCON
siIDH2
miR204-M
miR204-I
siIDH2
+ miR204-I
45 kDa

Fig.5B

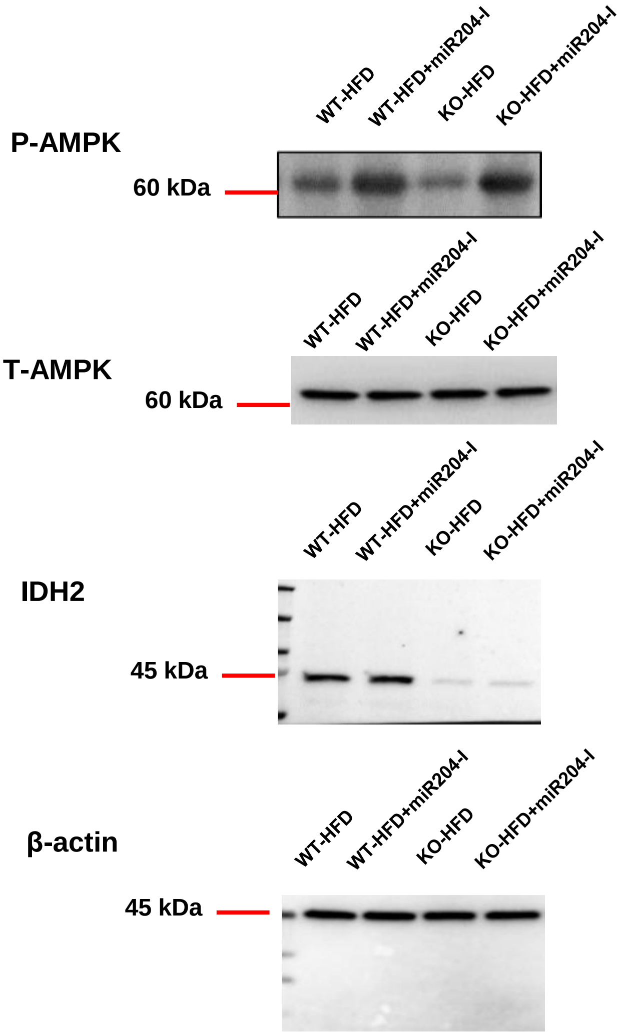
WT-HFD
WT-HFD+miR204-I
KO-HFD
KO-HFD+miR204-I
P-AMPK
60 kDa
WT-HFD
WT-HFD+miR204-I
KO-HFD
KO-HFD+miR204-I
T-AMPK
60 kDa
WT-HFD
WT-HFD+miR204-I
KO-HFD
KO-HFD+miR204-I
IDH2
45 kDa
WT-HFD
WT-HFD+miR204-I
KO-HFD
KO-HFD+miR204-I
β-actin
45 kDa

Fig.5B

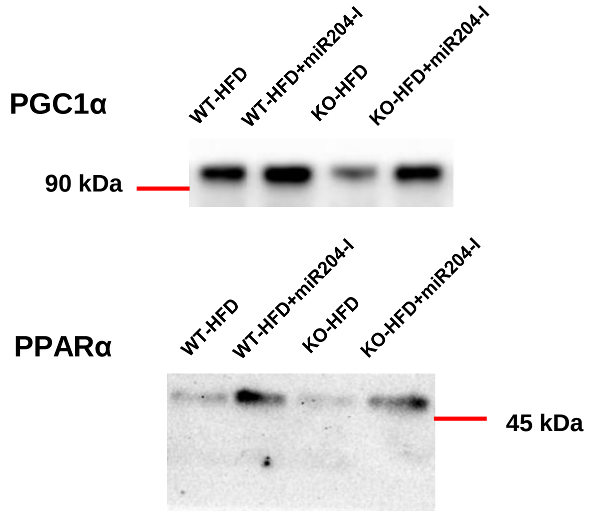
PGC1α
WT-HFD
WT-HFD+miR204-I
KO-HFD
KO-HFD+miR204-I
90 kDa
PPARα
WT-HFD
WT-HFD+miR204-I
KO-HFD
KO-HFD+miR204-I
45 kDa

Fig.6J

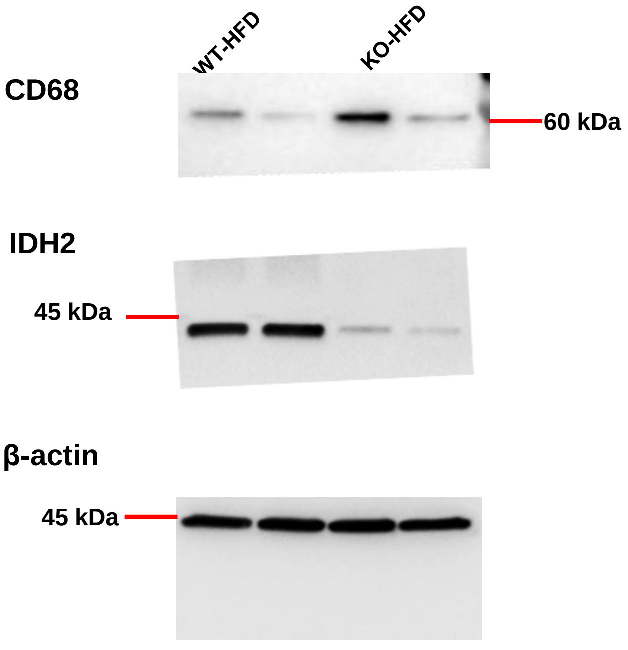
CD68
WT-HFD
KO-HFD
60 kDa
IDH2
45 kDa
β-actin
45 kDa
